# Thermally Regulated Methanol Selectivity in Photo‐Thermo Methane Oxidation Through Interfacial Methyl Transfer

**DOI:** 10.1002/advs.78386

**Published:** 2026-10-25

**Authors:** Youxun Xu, Chengzhi Guo, Chaoran Jiang, Leirun Chen, Juncong Wang, Enqi Chen, Yang Lan

**Affiliations:** ^1^ Department of Chemical Engineering University College London London UK; ^2^ SINOPEC (Beijing) Research Institute of Chemical Industry Co., Ltd Beijing China

**Keywords:** methane oxidation, methanol, photo‐thermo cascade catalysis, selectivity

## Abstract

The direct oxidation of methane to methanol has long been pursued as a low‐carbon alternative to syngas‐based production routes. However, its practical implementation remains constrained by low methanol selectivity in thermocatalysis and limited productivity in photocatalysis. Although temperature is intrinsic to catalytic systems, its role in photocatalysis has largely been regarded as secondary and predominantly kinetic. Here, we demonstrate that mild thermal input serves as an effective mechanistic lever to regulate reaction selectivity in a photo‐thermo cascade catalytic system based on Au nanoparticle‐decorated ZnO. This system achieves a methanol production rate of ∼800 µmol h^−1^ with 86% selectivity at 150°C, corresponding to an approximately tenfold increase in productivity and a 150% increase in selectivity relative to photocatalysis alone. While light irradiation enables methane C–H activation, thermal excitation reshapes the reaction network by promoting interfacial transfer of surface‐bound methyl species from oxide sites toward Au domains, thereby redirecting reaction flux toward methanol formation. Mechanistic studies support thermally promoted interfacial methyl transfer as a key contributor to the temperature‐regulated selectivity. This thermally tunable selectivity is further demonstrated across a series of Au‐loaded metal‐oxide semiconductors, highlighting interfacial pathway regulation as a general design principle for efficient and selective methane‐to‐methanol conversion.

## Graphical abstract

Photo‐thermo cascade catalysis enables highly selective methane‐to‐methanol conversion over Au‐ZnO, achieving a CH_3_OH production rate of ∼800 µmol h^−1^ with 86% selectivity. Thermal activation gates the transfer of methane‐derived methyl intermediates from ZnO to Au, thereby redirecting the reaction pathway toward methanol formation.

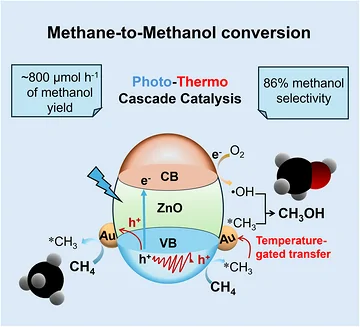

## 1 Introduction

Methanol is an emerging energy fuel and a key building block in the chemical industry, primarily used for producing formaldehyde, acetic acid, and plastics [1, 2, 3, 4, 5, 6]. The energy‐intensive conventional route for methanol synthesis in industry involves reforming methane, the principal component of natural gas, into syngas, followed by its conversion to methanol at high pressure (Figure S1) [7, 8]. This route accounts for ∼65% of global methanol production [6, 9]. The life‐cycle emissions from current methanol production and use are approximately 0.3 gigatonnes (Gt) of CO_2_ per year, accounting for about 10% of total emissions from the chemical sector. Therefore, it is of great importance to develop new routes that bypass synthesis gas for methanol synthesis, i.e. the direct oxidation of methane to methanol under mild conditions, to reduce the carbon emissions.

The direct oxidation of methane to methanol is attractive yet fundamentally challenging due to strong C‐H bonds (439 kJ mol^−1^), negligible electron affinity, and low polarisability [10, 11, 12, 13]. Many heterogeneous catalysts have been developed for the selective oxidation of methane to methanol by thermocatalysis, including isolated Rh‐anchored zeolite, AuPd colloids, and AuPd embedded zeolite [8, 14, 15]. However, the activation of methane typically necessitates expensive promoters, such as CO and H_2_O_2_ [6]. In‐situ synthesized H_2_O_2_ from H_2_ and O_2_ for methane oxidation offers an attractive alternative, yet showing moderate methanol production rate [15]. Recently, the direct oxidation of methane to oxygenates in the absence of CO and H_2_O_2_ under mild temperatures (< 200°C) was reported over Au‐supported ZSM‐5 and edge‐rich MoS_2_. However, the production rates remain extremely low (< 2 µmol h^−1^) with a selectivity of less than 40% [16, 17]. This is because thermal catalysis struggles to overcome this energy barrier for activation of the first C‐H bond under mild temperatures. In contrast, photocatalysis can readily achieve this through photo‐generated holes or photo‐generated reactive oxygen species (e.g. •OH radicals) [13, 18]. A variety of cocatalyst‐loaded metal oxides, such as Ag‐modified (001) dominated TiO_2_, Au nanoparticles modified In_2_O_3_, Au modified ZnO, AuPd modified ZnO [19, 20, 21, 22], have been reported for the selective oxidation of methane to methanol via photocatalysis only. However, the methanol production rate is still moderate (< 100 µmol h^−1^) and by‐products such as HCHO and/or CH_3_OOH are often formed in addition to methanol [20, 21, 22, 23, 24, 25, 26]. Therefore there is a clear trade‐off between the high selectivity and the high yield of methanol in conventional photocatalysis.

To enhance the yield of methane to methanol conversion, the introduction of thermocatalysis under mild conditions is essential due to its intrinsic fast reaction kinetics. More importantly, purely photocatalytic processes are primarily governed by the surface distribution of photogenerated charge carriers, which makes precise control over reaction pathways and product selectivity inherently challenging. In contrast, thermal input can actively modulate the adsorption–desorption behavior and spatial distribution of surface intermediates, thereby redirecting reaction pathways and improving selectivity. Therefore, we envisage that an ideal strategy is a photo‐thermo cascade process, in which light irradiation facilitates the initial C‐H activation of methane to generate key surface intermediates, while mild thermal input promotes their diffusion/spillover and site‐to‐site transfer to selectively reactive domains. For example, migration or “spillover” of methyl species between oxide supports and metal sites has been shown to be crucial for target product formation while suppressing undesired side reactions [27, 28]. Here, CH_3_* denotes a surface‐bound methyl species, distinct from freely diffusing •CH_3_. Thermally promoted interfacial transfer of CH_3_* between oxide and metal‐containing sites could therefore contribute to improved methanol selectivity and productivity under coupled light and thermal driving forces.

Here, we demonstrate highly efficient and selective production of CH_3_OH from methane by photo‐thermo cascade catalysis over Au nanoparticle‐modified metal‐oxide semiconductors. The Au nanoparticle‐decorated ZnO catalyst (Au‐ZnO) shows a methanol production rate of ∼800 µmol h^−1^ with 86% selectivity at 150°C. Under the present conditions, the charge‐characterization results support preferential photohole accumulation at Au‐related sites. Thermally promoted interfacial transfer of surface‐bound CH_3_* from ZnO toward Au‐containing domains is proposed as a contributor to the enhanced CH_3_OH selectivity. This photo‐thermo cascade strategy is effective for selective methane‐to‐methanol conversion across a range of Au‐loaded metal‐oxide semiconductors.

## 2 Results and Discussion

### 2.1 Performance of Methane Oxidation to Methanol

Photo‐thermo cascade catalytic methane oxidation reactions were conducted in a 230 mL custom‐made batch reactor (Figure S2). Different metal nanoparticles were first synthesized by the NaBH_4_ reduction method with PVP as a stabilizer and then loaded on ZnO. The numeric prefixes in the catalyst names denote nominal metal loadings in wt.%, calculated relative to the support mass. As shown in Figure 1a, HCHO is the primary product over pristine ZnO with a production rate of 565.2 µmol h^−1^ and methanol selectivity of less than 5%. After loading nominal 2 wt.% metal nanoparticles, the selectivity of CH_3_OH increases in the order of ZnO < Ag‐ZnO < Pt‐ZnO < Ru‐ZnO < Pd‐ZnO < Au‐ZnO. Au‐ZnO shows the highest selectivity of 74.2% with the highest production rate of CH_3_OH (642.8 µmol h^−^^1^). In addition, a small amount of C_2_H_6_ is produced. The influence of the Au loading amount was then investigated at 150°C (Figure 1b). Loading a small amount of Au (nominal 0.5 wt.%) on ZnO markedly changes the selectivity from HCHO to CH_3_OH. Further increasing the Au loading decreases the HCHO production rate, and almost no other oxygenates (CH_3_OOH and HCHO) are produced at nominal Au loadings of 3 and 5 wt.%. Considering the small difference in activity with further increases in Au loading, nominal 2 wt.% was chosen to balance performance and cost (hereafter 2Au–ZnO). The relevant NMR spectra and GC chromatogram are shown in Figures S3 and S4. CH_3_OH production and selectivity over Au‐ZnO with different Au loadings at 30°C show a similar trend (Figure S5). Notably, the overall product formation changes much less markedly than the product distribution, indicating that Au loading primarily regulates reaction‐pathway branching rather than proportionally controlling the total reaction flux. At higher Au loadings, the increased C_2_H_6_ formation is consistent with an increased contribution from a competing C–C coupling pathway.

**FIGURE 1:**
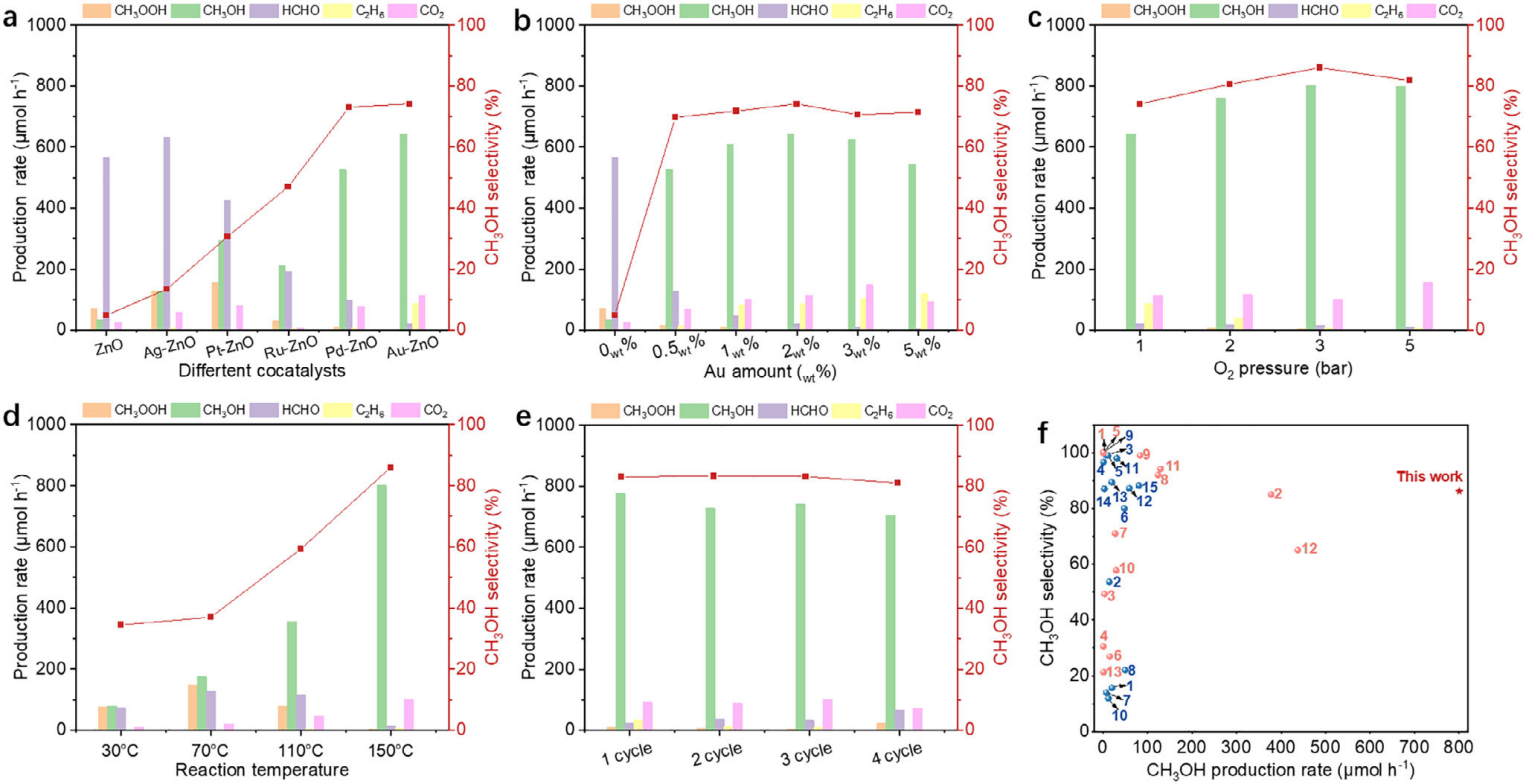
Photo‐thermo cascade catalytic methane oxidation to methanol. (a) Production rates of methane oxidation products over ZnO loaded with different metal nanoparticles (nominal 2 wt.%) and CH_3_OH selectivity at 150°C with 1 bar O_2_ and 20 bar CH_4_. (b) CH_3_OH production rate and selectivity over Au‐ZnO with different nominal Au loadings at 150°C with 1 bar O_2_ and 20 bar CH_4_. (c) Effect of O_2_ partial pressure over 2Au‐ZnO at a fixed CH_4_ pressure of 20 bar. (d) Influence of reaction temperature on product formation rates and CH_3_OH selectivity over 2Au‐ZnO. (e) Stability of 2Au‐ZnO over four cycles (0.5 h per cycle); 10 mg recovered catalyst was weighed for each subsequent cycle and reacted with fresh water after recharging 3 bar O_2_ and 20 bar CH_4_. Standard reaction conditions: 10 mg catalyst, 200 mL H_2_O, 3 bar O_2_ + 20 bar CH_4_, 365 nm LED (75 mW cm^−2^), 150°C, and 0.5 h. (f) Summary of CH_3_OH production rate and selectivity over reported benchmark catalysts. The blue data points represent photocatalysts: (1) Au/ZnO [29], (2) Au‐CoO_x_/TiO_2_ [30], (3) Au/ZnO [21], (4) q‐BiVO_4_ [31], (5) Au_1_/BP [32], (6) Ag‐TiO_2_(001) [19], (7) Ni‐NC‐TiO_2_ [33], (8) AuCu‐ZnO [34], (9) ZnO/Fe_2_O_3_ [10], (10) Pd‐def‐In_2_O_3_ [35], (11) PMOF–RuFe(OH) [36], (12) Co_1_/PCN [37], (13) AuNPs/In_2_O_3_ [20], (14) Ga_2_O_3_ [38], and (15) Au‐Pd/ZnO [22]. The red data points represent thermal catalysts: (1) Pd/CsPMA‐H [39], (2) Cu–Fe/ZSM‐5 [40], (3) AuPd/TiO_2_ [41], (4) Au‐ZSM‐5 [16], (5) Rh‐ZSM‐5 [14], (6) Au‐Pd colloid [8], (7) SAs Rh‐CeO_2_ NWs [42], (8) AuPd@ZSM‐5‐C16 [15], (9) Rh/TiO_2_ [43], (10) PdCu/zeolite [44], (11) Au/H‐MOR [45], (12) Rh_1_/pMOF [46], and (13) MoS_2_ [17].

To examine whether Au mainly promotes sequential HCHO‐to‐CH_3_OH conversion, HCHO‐feeding controls were performed (Figures S6 and S7). No CH_3_OH was detected from HCHO alone over either ZnO or 2Au‐ZnO, and adding HCHO under standard methane‐oxidation conditions did not increase CH_3_OH formation, arguing against released or solution‐phase HCHO as the major productive precursor to methanol.

Figure 1c shows the influence of O_2_ partial pressure on the production rate and selectivity of CH_3_OH. Increasing O_2_ pressure from 1 to 3 bar promotes CH_3_OH formation, whereas 5 bar increases CO_2_ formation and lowers CH_3_OH selectivity. These non‐monotonic changes indicate that O_2_ availability primarily regulates the competition among methyl coupling, selective oxygenation, and over‐oxidation pathways rather than proportionally controlling the overall reaction flux (Figure S8). Control experiments in the absence of light, methane, or catalyst show negligible product formation at 150°C for 1 h (Figure S9), confirming that methane is the carbon source and that light irradiation is essential for measurable product formation under these conditions. The impact of temperature on methanol production and selectivity was then examined over the optimized 2Au‐ZnO (Figure 1d). As the reaction temperature increases from 30°C to 150°C, the overall formation of observable products increases by approximately fourfold. At 30°C, the products are a mixture of CH_3_OOH, CH_3_OH, and HCHO with a CH_3_OH selectivity of 34.4%. The CH_3_OH selectivity increases to 37.0% at 70°C and 59.3% at 110°C, and the CH_3_OH production rate reaches 800.8 µmol h^−1^ with 86.0% selectivity at 150°C, with negligible HCHO and a small amount of CO_2_ produced as by‐products. These results show that elevated temperature promotes product formation while strongly shifting selectivity toward methanol over 2Au‐ZnO. Matched temperature‐dependent measurements over bare ZnO show that heating also strongly increases the overall oxygenate formation but leaves the reaction predominantly HCHO‐selective, indicating that temperature generally accelerates the illuminated ZnO reaction network, whereas the pronounced temperature‐dependent shift toward CH_3_OH is Au‐dependent (Figure S10).

Figure S11 shows the time‐dependent product amounts and CH_3_OH selectivity. The interval‐average CH_3_OH production rate decreases as CH_3_OH accumulates in solution and undergoes competing secondary conversion (Figure S12). Controls with methanol in the feed confirm that methanol concentration influences the net methane‐oxidation product distribution (Figure S12 and Table S1). Similar trends have been reported in thermocatalytic methane oxidation [15]. The stability test of 2Au‐ZnO is shown in Figure 1e. The catalyst maintained its performance over four cycles without a significant decrease in CH_3_OH production rate or selectivity; 10 mg recovered catalyst was used for each subsequent cycle with fresh water and renewed gas feed. XPS and TEM analyses show that the Au chemical state and particle‐size distribution remain similar after reaction (Figure 2c,d and Figures S13 and S14). Figure 1f compares Au‐ZnO with reported photocatalytic and thermocatalytic systems. Au‐ZnO produces ∼400 µmol CH_3_OH with >85% selectivity in 0.5 h, approximately one order of magnitude above the production reported for prior photocatalysts and above thermal systems using CO as a reductant or H_2_O_2_ as an oxidant [8, 14, 15].

**FIGURE 2:**
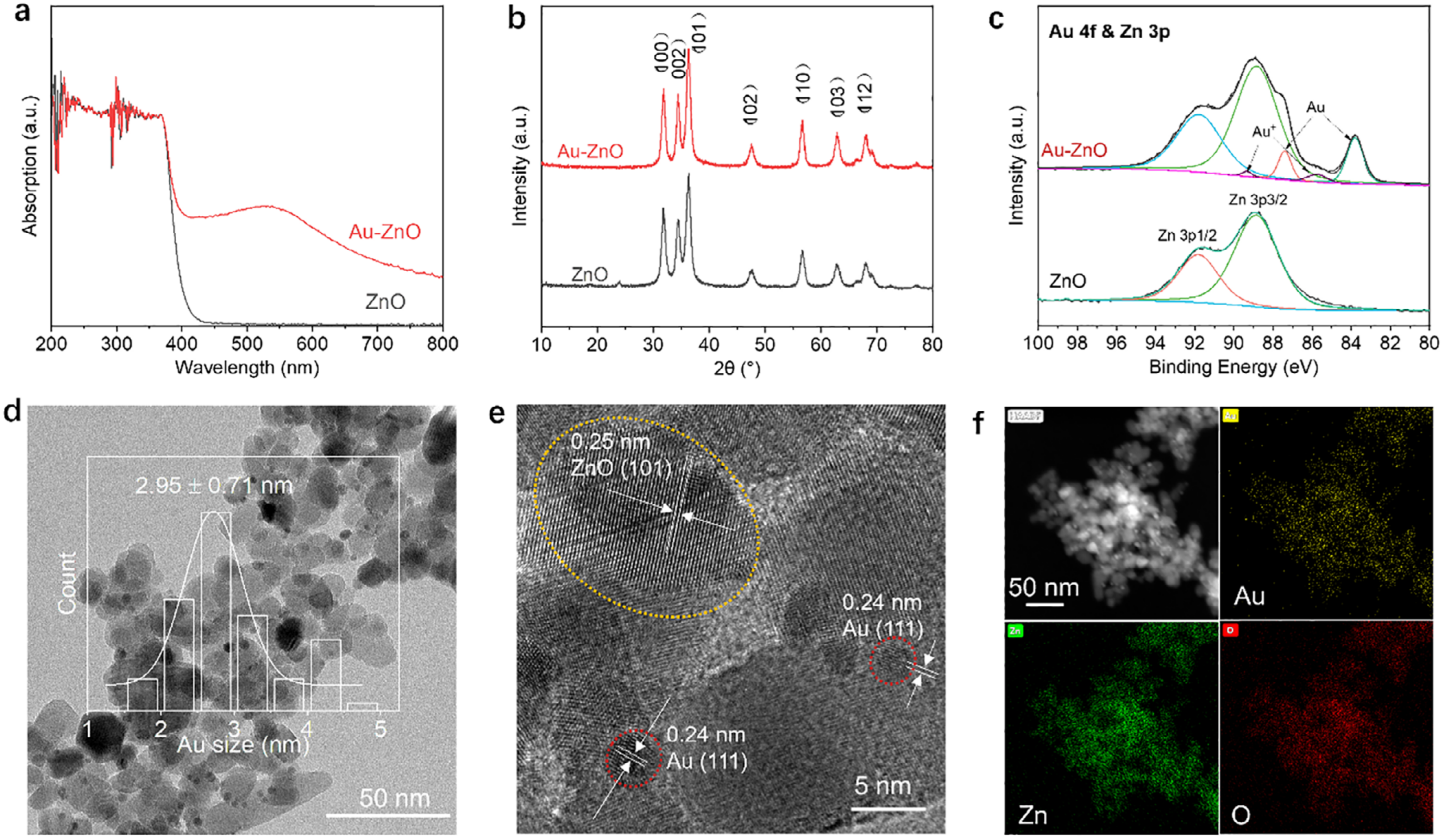
Characterization of the photocatalysts. (a) UV‐Vis spectra and (b) XRD patterns of ZnO and Au‐ZnO. (c) Au 4f and Zn 3p high‐resolution XPS spectra of ZnO and Au‐ZnO. (d) TEM image, (e) high‐resolution TEM image and (f) element mappings of Au‐ZnO. The inset of Figure 2d shows the size distribution of the Au nanoparticles. The yellow circle in Figure 2e highlights the ZnO particles and the red circles highlight Au nanoparticles.

### 2.2 Characterization of the Photocatalysts

The photon‐absorption properties of the catalysts were analysed using ultraviolet‐visible (UV‐Vis) diffuse reflectance spectroscopy. ZnO shows an absorption edge at 400 nm, which corresponds to an optical bandgap of 3.1 eV (Figure 2a). A pronounced absorption throughout the visible spectrum is noticeable over Au‐ZnO, which is probably due to the scattering effect of Au [47]. The strong absorption at 530 nm originates from localised surface plasmon resonance of Au nanoparticles with the light [48]. The X‐ray diffraction (XRD) pattern of ZnO reveals that all peaks correspond to the hexagonal wurtzite phase (JCPDS # 99–0111) of ZnO (Figure 2b). No new peaks for Au are discernible over Au‐ZnO due to the high dispersion and small particle sizes of Au (as shown later in the TEM analysis) [49]. XPS analysis was performed to further investigate the chemical states of Au nanoparticles on ZnO (Figure 2c). The two peaks at 88.8 eV and 91.8 eV of ZnO correspond to Zn 3p_3/2_ and 3p_1/2_, respectively [34]. There are two peaks located at 83.8 eV and 87.4 eV with Δ_BE_ = 3.6 eV, corresponding to the Au^0^ 4f_7/2_ and Au^0^ 4f_5/2_, respectively [49, 50]. The two small peaks located at 85.8 eV (shifted by 2 eV from the Au^0^ 4f_7/2_ state) and 89.4 eV are ascribed to the Au^+^ 4f_7/2_ and Au^+^ 4f_5/2_, respectively [50]. The above analysis suggests that the Au nanoparticle on ZnO is mainly in the Au^0^ state with a small proportion of oxidised Au. TEM was conducted to examine the morphology of the photocatalysts. ZnO exhibits an average particle size of 10–20 nm and Au nanoparticles are well dispersed on the ZnO surface with an average particle size of ∼3 nm (Figure 2d). A high‐resolution TEM image shows that the interplanar lattice spacing of 0.25 nm corresponds to the (101) plane of ZnO and the interplanar lattice spacing of 0.24 nm corresponds to the (111) plane of Au nanoparticles. Energy dispersive spectrometry (EDS) mapping in Figure 2f shows that Au is uniformly dispersed on the ZnO support.

#### 2.2.1 Reaction Mechanism

To obtain insights into the role of Au in charge transfer and recombination, photoluminescence (PL) spectroscopy was employed. The PL peak intensity of Au–ZnO substantially diminishes compared with that of ZnO, suggesting reduced charge recombination (Figure 3a). Electrochemical characterizations show a higher photocurrent density over Au‐ZnO in CH_4_‐saturated electrolyte and a lower onset potential for methane oxidation (Figure 3b,c), indicating altered charge separation and oxidation behavior under these characterization conditions. Upon light irradiation, the Au 4d_3/2_ and Au 4d_5/2_ peaks shift by approximately 0.3 eV toward higher binding energy, indicating a more electron‐deficient Au‐related electronic environment and supporting preferential photohole accumulation at Au‐related sites under the present conditions [34, 49]. Because charge accumulation at Au‐semiconductor interfaces depends on the local interfacial structure and reaction conditions [51], this assignment is system‐specific. EPR measurements show stronger DMPO‐OOH and DMPO‐OH signals over Au‐ZnO than over ZnO (Figure 3e,f). These characterization results indicate altered charge‐transfer and ROS‐generation behavior, but do not imply a proportional increase in reactor‐level oxygenate productivity.

**FIGURE 3:**
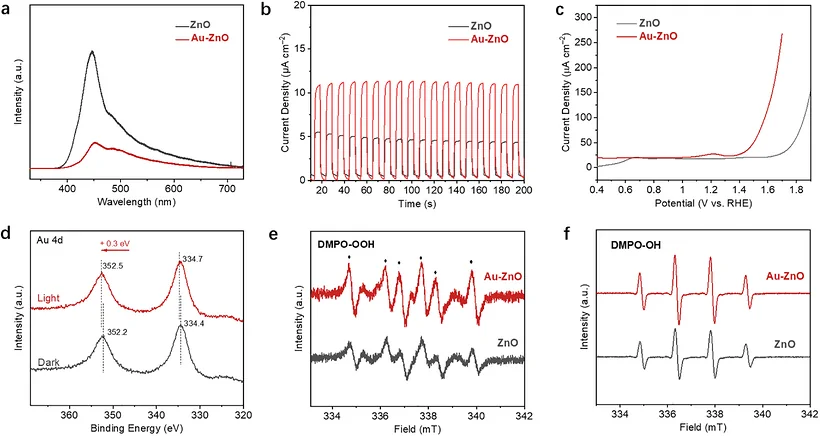
Charge transfer. (a) PL spectra of ZnO and Au‐ZnO. (b) Transient photocurrent density curves with a bias of 0.6 V vs. RHE and (c) electrochemical oxidation of methane over ZnO and Au‐ZnO. (d) In situ XPS spectra of Au 4d under dark and light irradiation conditions over Au‐ZnO. (e) EPR spectra of ZnO and Au‐ZnO in air‐saturated methanol solution under light irradiation for the detection of •OOH. (f) EPR spectra of ZnO and Au‐ZnO for the detection of •OH radicals.

As the activation of methane molecules is the most crucial step in selective methane oxidation, temperature‐programmed desorption (TPD) of CH_4_ was used to compare methane adsorption environments on ZnO and Au‐ZnO (Figure 4a). For ZnO, a low‐temperature desorption peak is observed at 173°C; this feature shifts to 115°C and broadens over Au‐ZnO. This change is consistent with additional weakly bound methane populations associated with Au‐related and/or Au‐ZnO interfacial sites [52]. The high‐temperature desorption peak shifts from 401°C on ZnO to 420°C on Au‐ZnO, indicating a change in the strongly adsorbed methane population [53]. These differences show that Au modifies methane adsorption/desorption environments and the distribution of surface binding sites under the TPD conditions.

**FIGURE 4:**
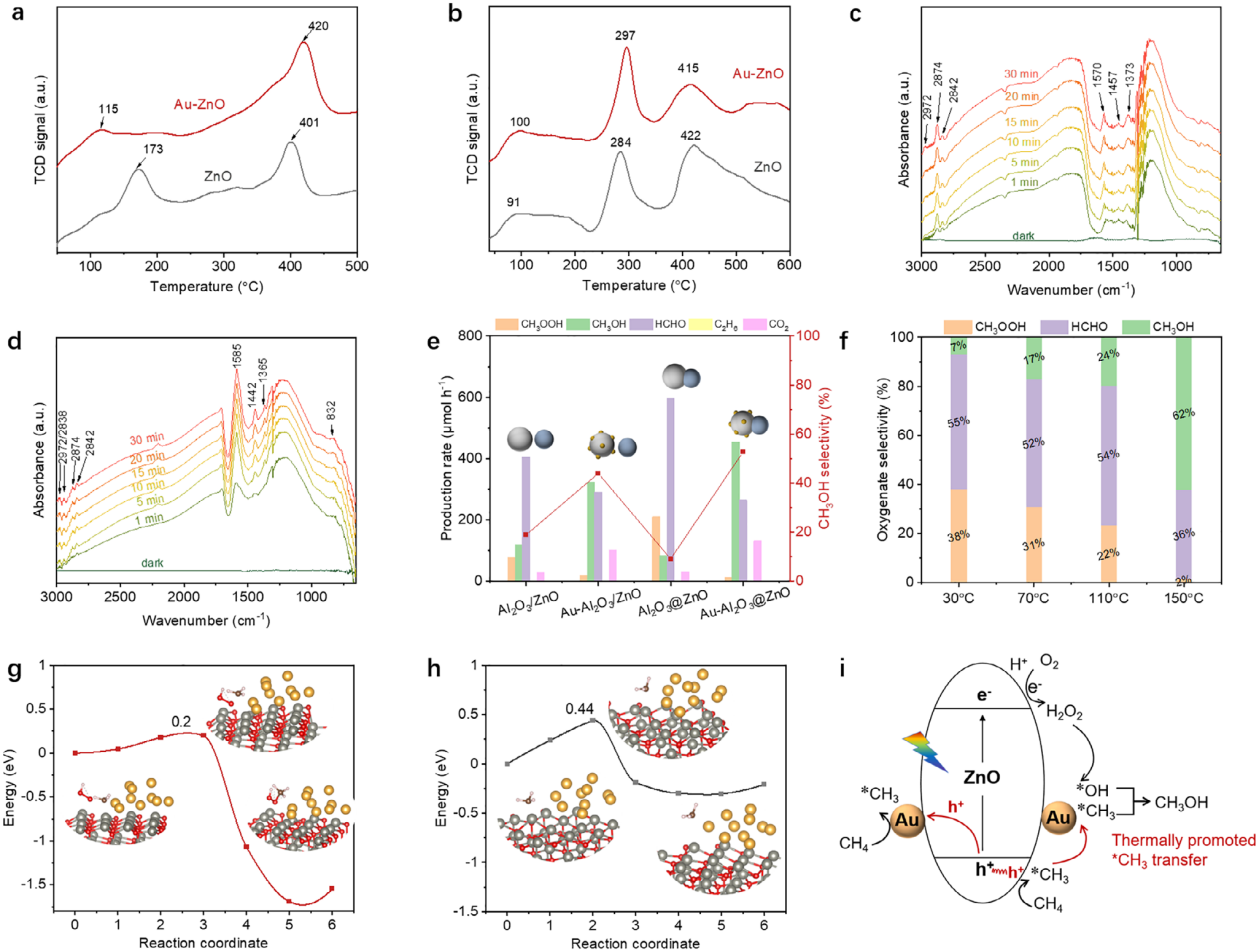
Mechanistic studies of methane oxidation to methanol. TPD profiles of (a) CH_4_ and (b) CH_3_OH over ZnO and 2Au‐ZnO. In situ DRIFTS for methane oxidation under light irradiation over (c) ZnO and (d) 2Au‐ZnO at room temperature in the presence of CH_4_, O_2_, and water vapour (CH_4_:O_2_ volume ratio = 20:3). (e) Photo‐thermo methane oxidation over Al_2_O_3_@ZnO, Au‐Al_2_O_3_@ZnO, Al_2_O_3_/ZnO, and Au‐Al_2_O_3_/ZnO. The inset illustrates the catalyst architectures. Grey, blue, and yellow spheres represent Al_2_O_3_, ZnO, and Au particles, respectively. Al_2_O_3_@ZnO denotes the Al_2_O_3_‐ZnO composite. Au‐Al_2_O_3_@ZnO indicates that Au nanoparticles are loaded only on the Al_2_O_3_ domain of Al_2_O_3_@ZnO. Al_2_O_3_/ZnO and Au–Al_2_O_3_/ZnO denote simultaneous introduction of the corresponding separate particles. Reaction conditions: 20 mg catalyst, 200 mL H_2_O, 3 bar O_2_ + 20 bar CH_4_, 365 nm LED (75 mW cm^−2^), 150°C, and 0.5 h. (f) CH_3_OH selectivity over Au‐Al_2_O_3_@ZnO at different reaction temperatures. Calculated energy profiles for (g) coupling of surface‐bound CH_3_* with a radical‐like •OOH species at the ZnO interface and (h) transfer of surface‐bound CH_3_* from ZnO to an Au interfacial site. Grey, red, yellow, brown, and white spheres represent Zn, O, Au, C, and H atoms, respectively. (i) Proposed reaction mechanism over 2Au‐ZnO, including thermally promoted CH_3_* transfer. CH_3_* denotes a surface‐bound methyl species and is distinct from a free •CH_3_ radical.

The CH_3_OH‐TPD profiles show distinct methanol‐derived desorption behavior on ZnO and Au‐ZnO (Figure 4b and Figure S15). Au‐ZnO shows a lower‐intensity low‐temperature methanol feature and a suppressed high‐temperature tail relative to ZnO. These differences indicate altered methanol adsorption/desorption and high‐temperature evolution under the He‐TPD protocol, rather than in situ desorption kinetics or a specific deep‐oxidation threshold under reaction conditions.

In situ diffuse reflectance infrared Fourier transform spectroscopy (DRIFTS) under light irradiation was used to examine methane‐derived surface species (Figure 4c,d). For ZnO, bands at 2900–3000 and 2800–2900 cm^−1^, and at 1457 and 1373 cm^−1^, occur in C–H stretching and bending regions associated with methane‐derived species [10]. Upon introduction of Au, the band near 2874 cm^−1^ decreases, while the 1457 and 1373 cm^−1^ features increase and shift to lower wavenumbers, indicating that Au changes the population and/or environment of surface intermediates. A band near 832 cm^−1^ is consistent with C–C‐containing species, in line with detection of ethane as a by‐product. The 1570 cm^−1^ region is consistent with OCO/formate‐like species but is not treated as a unique fingerprint because overlapping contributions are possible [54]. These spectra support altered intermediate populations over Au‐ZnO without directly proving methyl transfer.

Temperature‐resolved DRIFTS shows pronounced time‐dependent growth near 1570 and 1370 cm^−1^ on ZnO at 150°C, whereas the corresponding growth is much weaker on Au‐ZnO (Figure S16). Because these regions contain overlapping contributions, they are used only as comparative markers, supporting an Au‐dependent redistribution of methane‐derived surface intermediates at elevated temperature.

The Al_2_O_3_‐based experiments were designed as spatial/proximity controls rather than as an electronic analogue of ZnO. Al_2_O_3_ and Au‐Al_2_O_3_ exhibit negligible methane‐oxidation activity (Figure S17). When Al_2_O_3_ and ZnO particles are introduced simultaneously (Al_2_O_3_/ZnO), HCHO remains the main product and CH_3_OH selectivity is below 19% (Figure 4e), similar to ZnO alone. When physically mixed Au‐Al_2_O_3_ and ZnO particles are introduced (Au‐Al_2_O_3_/ZnO), CH_3_OH selectivity increases to approximately 44%. Closer integration in Au‐Al_2_O_3_@ZnO further increases CH_3_OH selectivity to approximately 53%. The CH_3_OH selectivity of Au‐Al_2_O_3_@ZnO also increases from 7% to 62% between 30°C and 150°C (Figure 4f and Figure S18). Together, these spatial controls indicate functional cooperation between spatially distinct ZnO and Au‐containing domains.

To examine the temperature‐dependent pathway branching, DFT calculations were performed for surface‐bound CH_3_* at the Au‐ZnO interface. CH_3_* preferentially binds to Zn sites on ZnO (Figure S19). Coupling of CH_3_* with a radical‐like •OOH species has a calculated barrier of 0.20 eV (Figure 4g), whereas direct coupling with adsorbed OOH* in the distinct configuration shown in Figure S20 has a barrier of 1.44 eV. Transfer of CH_3_* from a Zn site to an Au edge site has a calculated barrier of 0.44 eV (Figure 4h). These calculations establish a physically accessible interfacial‐transfer pathway for a surface‐bound methyl species.

The 0.44 eV CH_3_* transfer step is more temperature‐sensitive than the 0.20 eV C–O coupling step in a simple Arrhenius comparison (Figure S21). This comparison is used only qualitatively, because differences in prefactors, surface coverages, and reactant configurations prevent prediction of an absolute branching ratio or rate crossover.

The electron‐transfer process involved in O_2_ reduction and H_2_O_2_ formation was also examined (Figure S22). Based on the above analysis, the mechanism shown in Figure 4i is proposed. Light generates the reactive charge carriers, while mild heating increases the kinetic accessibility of interfacial transfer of surface‐bound CH_3_* toward Au‐containing domains. This transfer is proposed as one contributor to the Au‐dependent methanol selectivity.

The broader influence of thermally regulated methanol selectivity was further investigated over a series of metal‐oxide semiconductors loaded with Au nanoparticles (TiO_2_, CeO_2_, In_2_O_3_, BaTiO_3_, and SrTiO_3_) by photo‐thermo cascade catalysis (Figure 5a). For the pure metal‐oxide semiconductors, the main products are CH_3_OOH or HCHO, with CH_3_OH selectivities of 22%, 40%, 20%, 23%, and 45% over TiO_2_, CeO_2_, In_2_O_3_, BaTiO_3_, and SrTiO_3_, respectively. Upon loading with Au nanoparticles, the CH_3_OH selectivity increases to 66%, 76%, 73%, 76%, and 79% over Au‐TiO_2_, Au‐CeO_2_, Au‐In_2_O_3_, Au‐BaTiO_3_, and Au‐SrTiO_3_, respectively. This enhancement highlights the recurring role of Au‐containing domains in steering methane oxidation toward methanol across diverse oxide supports. Under photocatalytic conditions at 30°C, the CH_3_OH selectivity increases from 0% for CeO_2_ and 7% for TiO_2_ to 33% for 5Au‐CeO_2_ and 43% for 5Au‐TiO_2_, respectively (Figures S23 and S24), consistent with the results observed for Au‐ZnO. Under photo‐thermo cascade conditions, both the production and selectivity of methanol increase as the reaction temperature rises over 2Au‐CeO_2_ and 2Au‐TiO_2_ (Figure 5b,c). The recurring Au‐dependent shift toward CH_3_OH across these oxide supports indicates the broader relevance of temperature‐sensitive pathway regulation involving Au‐containing domains.

**FIGURE 5:**
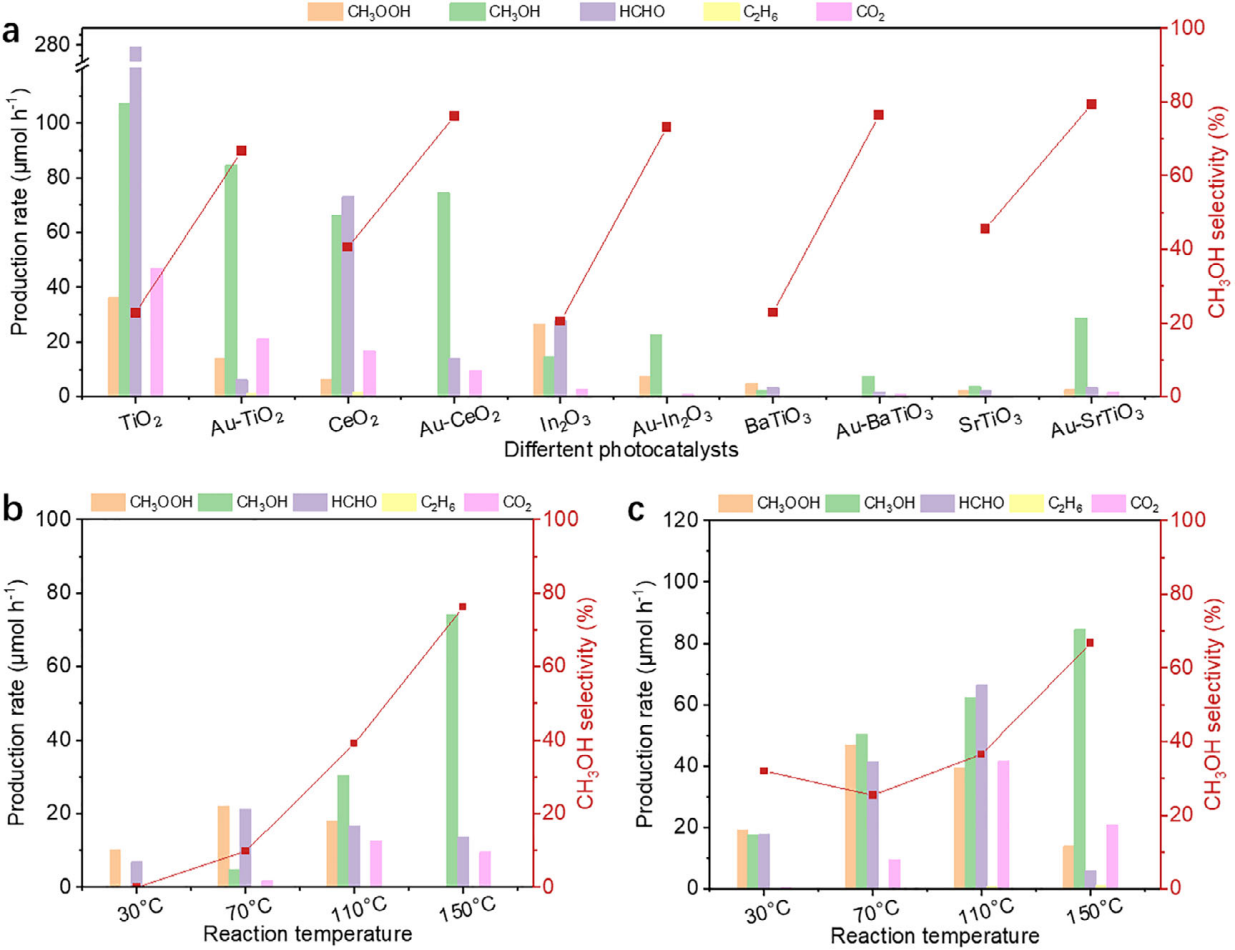
Photo‐thermo cascade catalytic methane oxidation over other metal‐oxide semiconductors. (a) Methane‐oxidation products and CH_3_OH selectivity over Au‐loaded TiO_2_, CeO_2_, In_2_O_3_, BaTiO_3_, and SrTiO_3_. Influence of reaction temperature on the production and selectivity of CH_3_OH over (b) Au‐CeO_2_ and (c) Au‐TiO_2_. Standard reaction conditions: 10 mg photocatalyst, 200 mL H_2_O, 3 bar O_2_ + 20 bar CH_4_, 365 nm LED (75 mW cm^−2^), 150°C, and 0.5 h.

#### 2.2.2 Conclusions

In summary, this work reports a benchmark methanol productivity with high selectivity from methane oxidation via photo‐thermo cascade catalysis. With the synergistic effect of light and thermal input, a high methanol production rate of ∼800 µmol h^−1^ (80 mmol g^−1^ h^−1^) with 86% selectivity is achieved over Au nanoparticle‐decorated ZnO, approximately ten times higher than that driven by photocatalysis alone. The combined catalytic and mechanistic results support an Au‐dependent redistribution of methane‐derived intermediates, with thermally promoted interfacial transfer of surface‐bound methyl species proposed as a contributor to the enhanced CH_3_OH selectivity. This photo‐thermo cascade strategy is effective across a series of Au‐loaded metal oxides, providing a promising approach for selective methane‐to‐methanol conversion.

## Author Contributions

Y.L. supervised the progress of the whole project. Y.X. and C.G. proposed the idea, designed and conducted experiments, analysed data and drafted the article. C.J., L.C., and J.W. contributed to the discussion of the mechanism and the revision of the article. E.C. conducted the in situ XPS test and participated in the revision of the article.

## Conflicts of Interest

The authors declare no conflicts of interest.

## Supporting information




**Supporting File**: advs78386‐sup‐0001‐SuppMat.docx

## Data Availability

The data that support the findings of this study are available from the corresponding author upon reasonable request.
