# Supplementary material for: Thermally Regulated Methanol Selectivity in Photo‐Thermo Methane Oxidation Through Interfacial Methyl Transfer
Source: Adv Sci (Weinh). 2026 Oct 25:e78386. Online ahead of print. doi: 10.1002/advs.78386 (PMC13696773; doi:10.1002/advs.78386)
Supplement: Supplementary file 1 [file ADVS-9999-e78386-s001.docx]

**Supporting Information for**

# Thermally Regulated Methanol Selectivity in Photo-Thermo Methane Oxidation through Interfacial Methyl Transfer

**Authors:** Youxun Xu^1,2 +^, Chengzhi Guo^1 +^, Chaoran Jiang^2^, Leirun Chen^1^, Juncong Wang^1^, Enqi Chen^1^ and Yang Lan^1^*

**Affiliations:**

1. Department of Chemical Engineering, University College London, London WC1E 7JE, U.K.
2. SINOPEC (Beijing) Research Institute of Chemical Industry Co., Ltd., Beijing 100013, China

*Correspondence to: yang.lan@ucl.ac.uk

^+^Y.X. and C.G. contributed equally to this work.

## Methods

### Chemicals and materials

All reagents were obtained from commercial sources and used as received. The methane (CP grade), oxygen, and argon (zero grade) were supplied by BOC. Zinc nitrate hexahydrate (98%, extra pure) and oxalic acid (98%, anhydrous) were purchased from Acros Organics. Gold (III) chloride trihydrate (≥ 99.9% trace metals basis), potassium tetrachloropalladate (98%), chloroplatinic acid hexahydrate, silver nitrate, cerium (IV) oxide (nanopowder, < 25 nm), indium (III) oxide (nanopowder, < 100 nm), strontium titanate (nanopowder, < 100 nm), barium titanate (nanopowder, < 100 nm), polyvinylpyrrolidone (PVP, average molecular weight ~55,000) and sodium borohydride (≥ 98.0%) were obtained from Sigma Aldrich. Ruthenium (III) chloride hydrate (99.9%, Ru 38% min.) was obtained from Alfa Aesar. Aluminum oxide (99.7+%, extra pure) was purchased from fisher scientific. Pierce 16% (w/v) methanol-free formaldehyde in water (Thermo Scientific, Cat. No. 28908) was used for the HCHO-feeding controls. According to the manufacturer's product specification, this stock is prepared from paraformaldehyde and supplied methanol-free.

**Material synthesis**

The zinc oxide (ZnO) nanoparticles were synthesised using the precipitation method, with slight modifications to the process described in the literature.^[1]^ Zinc nitrate (Zn(NO_3_)_2_) and oxalic acid, each at a molar concentration of 0.025 moles, were dissolved separately in 500 mL of deionized water. Subsequently, the oxalic acid solution was slowly added to the zinc nitrate solution drop by drop using a separating funnel, leading to the formation of zinc oxalate precipitates. These precipitates were then oven-dried at 60 °C and later calcined in air at 350 °C for 6 h.

Au, Ag, Pt, Ru, and Pd nanoparticles were synthesised by NaBH_4_ reduction with PVP (average molecular weight ~55,000) as a stabiliser. The numeric prefixes in catalyst names denote nominal metal loadings in wt%, calculated relative to the support mass. For the Au-loading series, 200 mg of support was used in each preparation. A HAuCl_4_·3H_2_O stock solution (2.3 mg mL^-1^; approximately 1.15 mg Au mL^-1^) was used, and 0.87, 1.74, 3.48, 5.22, and 8.69 mL of stock solution were added to obtain nominal Au loadings of 0.5, 1, 2, 3, and 5 wt%, respectively, based on the support mass. The precursor solution was added to 80 mL water and stirred vigorously, and PVP was added at a PVP/metal mass ratio of 1/2. The solution was placed in an ice bath for 10 min before freshly prepared NaBH_4_ solution (0.1 M; NaBH_4_/total metal = 10/1, mol/mol) was added at 0.5 mL min^-1^. After reduction for 30 min, 200 mg support was added. For TiO_2_ (P90), CeO_2_, In_2_O_3_, BaTiO_3_, SrTiO_3_, and Al_2_O_3_, the colloidal suspension was adjusted to pH 1 with H_2_SO_4_ before addition of the support. After a further 30 min, the catalyst was collected by centrifugation, washed three times with distilled water, and dried at 60 °C overnight.

Al_2_O_3_@ZnO and Au-Al_2_O_3_@ZnO were synthesised by adding Al_2_O_3_ or Au-Al_2_O_3_ particles to the Zn(NO_3_)_2_ solution, followed by the addition of an oxalic acid solution to form zinc oxalate precipitates on the Al_2_O_3_ or Au-Al_2_O_3_ surface. These precipitates were then oven-dried at 60 °C and subsequently calcined in air at 350 °C for 6 h.

**Characterisations**

X-ray diffraction (XRD) was conducted using a Bruker D8 diffractometer with a Cu Kα source (λ=1.5418 Å, scanning a 2Theta range from 10 to 80°). Ultraviolet-visible diffuse reflectance spectroscopy (UV-Vis) measurements were performed using a Shimadzu UV-2550 spectrophotometer equipped with an integrating sphere, and the reflectance values were converted to absorption using the Kubelka-Munk equation. X-ray photoelectron spectroscopy (XPS) analysis was carried out with a Thermo K-alpha XPS system, utilising an Al Kα source with a photon energy (hν) of 1486.6 eV. Transmission electron microscopy (TEM) analysis was conducted using an FEI Talos F200X instrument. Photoluminescence (PL) data were collected from 330 nm to 800 nm using a Renishaw InVia spectroscope with a 325 nm laser as the excitation source. In-situ XPS measurements were conducted using an Axis Supra (Kratos Analytical Ltd., UK) system, equipped with an Al Kα X-ray source (1486.6 eV) and a 365 LED lamp as the light source.

**Electrochemical measurement**

The photoelectrodes were prepared using a spin-coating technique. In a typical procedure, 50 mg of the catalyst was dispersed in 5 mL of ethanol and sonicated for 60 minutes. Fluorine-doped tin oxide (FTO) glass substrates, each measuring 2 cm by 2 cm, were used. An 80 μL aliquot of the suspension was applied to the FTO glass and spun at 500 rpm for 30 seconds. Following the spin-coating process, the electrode was placed on a hotplate and dried at 100 °C for 15 minutes.

All electrochemical tests were performed in a three-electrode cell using a 0.5 M Na_2_SO_4_ solution as the electrolyte. The FTO glass coated with the catalyst acted as the working electrode, while a platinum plate served as the counter electrode and an Ag/AgCl electrode in a saturated KCl solution functioned as the reference electrode. The electrochemical oxidation of methane was conducted within a voltage range of 0.4 to 1.8 V vs. RHE at a scan rate of 20 mV s^-1^. For the transient photocurrent density, a bias potential of 0.6 V vs. RHE was applied. A 300 W Xenon (Xe) lamp was used as the light source, with the light being intermittently chopped every 5 seconds.

**Electron paramagnetic resonance (EPR) spectroscopy measurement**

EPR spectroscopy was performed using a Bruker E580 X-band spectrometer with 5,5-dimethyl-1-pyrroline-N-oxide (DMPO) as the trapping agent. For the •OOH test, 5 mg catalyst was dispersed in 5 mL air-saturated methanol containing 50 mM DMPO and illuminated with a 365 nm LED for 10 min at room temperature while stirring. A 0.5 mL aliquot was then transferred promptly to a quartz tube. The modulation frequency was 100 kHz, microwave frequency 9.46 GHz, sweep time 60 s, microwave output 10 mW, and scan range 330–345 mT. The dark control used the same procedure without irradiation. For the •OH test, water containing 10 mM DMPO was used. These spin-trapping measurements probe relative radical-generation capability under the stated conditions and are not calibrated measurements of reactive-oxygen-species flux under the pressurised methane-oxidation conditions.

**In situ DRIFTS measurement**

In situ DRIFTS was performed using a Nicolet iS51 FTIR spectrometer equipped with an MCT detector, Harrick cell, and Praying Mantis accessory. Each spectrum was obtained by averaging 128 scans at 4 cm^-1^ resolution. The cell contained two ZnSe windows for IR transmission and a quartz window for 365 nm irradiation. For each experiment, 50 mg catalyst was placed in the sample holder with a flat surface and purged for 30 min in the dark with water-vapour-saturated CH_4_/O_2_ (CH_4_:O_2_ = 20:3). The cell was then sealed. For the original room-temperature series in Figure 4c and 4d, spectra were acquired after 1, 5, 10, 20, and 30 min irradiation. For the comparative temperature-resolved series in Figure S16, the sample holder was controlled at 30 or 150 °C, a dark spectrum was recorded after equilibration at the corresponding temperature, and illuminated spectra were acquired after 1, 2, 5, 10, and 20 min. Difference spectra were calculated as light minus dark at the same temperature. Local peak heights near 1570 and 1370 cm^-1^ were obtained after local linear baseline correction. Because overlapping species can contribute to these regions, the values are used as comparative temporal markers rather than calibrated surface coverages or uniquely assigned intermediate concentrations.

**TPD measurement**

CH_4_-TPD was performed using Automatic Chemisorption Analyzer (BSD-Chem C200). 50 mg catalyst was placed in a reaction tube and subjected to a temperature program from room temperature to 500 °C at a rate of 10 °C min^–1^ for drying and pretreatment. The sample was then purged with a flow of helium gas (50 mL min^–1^) for 1 hour and subsequently cooled to 50 °C. Methane gas was introduced for 1 hour to achieve saturation. After methane exposure, the flow was switched back to helium gas (50 mL min^–1^) to purge for an additional hour, removing physically adsorbed methane from the surface. Finally, the temperature was increased to 500 °C at a rate of 10 °C min^–1^ under a helium atmosphere to desorb the gases, which were detected using a thermal conductivity detector (TCD).

For CH_3_OH-TPD, 100 mg catalyst was loaded into a quartz tube and pretreated under He flow (40 mL min^–1^) by heating to 300 °C at 10 °C min^–1^ and holding for 60 min, followed by cooling to 40 °C. Methanol adsorption was performed at 40 °C by switching the gas flow to He saturated with methanol vapor, generated by bubbling He through methanol maintained at 0 °C. After 45 min, adsorption saturation was confirmed by a stable m/z = 31 signal in the MS. The sample was then purged with pure He at 40 °C for 60 min until the m/z = 31 signal returned to baseline. Temperature-programmed desorption was carried out from 40 to 600 °C at 10 °C min^–1^ under He (40 mL min^–1^), with desorbed species monitored by TCD and MS. The temperature was held at 600 °C for 10 min before cooling.

**DFT calculations**

All density functional theory (DFT) calculations were performed using the Vienna *Ab initio* Simulation Package (VASP) with the projector augmented-wave (PAW) method.^[2,3]^ The exchange–correlation interactions were described using the generalized gradient approximation (GGA) with the Perdew-Burke-Ernzerhof (PBE) functional.^[4]^ A plane-wave energy cutoff of 450 eV was employed. Partial occupancies were treated using the Gaussian smearing method with a smearing width of 0.2 eV.

The ZnO (0001) surface was modelled by a slab consisting of four Zn layers, containing 64 Zn atoms per unit cell. A large surface supercell was constructed to accommodate an Au_10_ cluster supported on the ZnO (0001) surface. Due to the large size of the surface model, a Γ-point (1 × 1 × 1) *k*-point mesh was used for all surface calculations. The electronic self-consistent calculations were converged to 1 × 10^-5^ eV, and the geometry optimizations were performed until the residual forces on each atom were below 0.05 eV Å^-1^. Transition states for surface reactions and CH_3_* transfer were located using the climbing-image nudged elastic band (CI-NEB) method,^[5]^ with the same force convergence criterion of 0.05 eV Å^-1^.

### Catalytic experiments

Photo-thermo cascade methane oxidation was conducted in a 230 mL custom-made batch reactor with an integrated quartz window. Typically, 10 mg catalyst was dispersed in 200 mL water and sonicated for 10 min. The suspension was transferred to the reactor, which was purged with O_2_ for 15 min, pressurised at room temperature with 3 bar O_2_ and 20 bar CH_4_, and stirred at 500 rpm while heating. A sheathed thermocouple monitored the liquid temperature. Upon reaching the target temperature, the reactor was irradiated with a 365 nm LED (Beijing Perfect Light, PLS-LED 100C) at 75 mW cm^-2^ through a 12.56 cm^2^ window. After reaction, the reactor was cooled rapidly to ~25 °C. Gas products were sampled with a GC syringe and analysed using the BID-2010 Plus gas chromatograph (Shimadzu); quantified CO was treated as a C1 gas-phase product. The Figure S4 chromatogram was obtained using this BID configuration. Liquid products were filtered before analysis.

The concentrations of CH_3_OOH and CH_3_OH in the liquid phase were quantified using ^1^H NMR spectroscopy (Bruker Avance Neo 500 MHz). For this, 0.5 mL of the liquid product was mixed with 0.1 mL of D_2_O containing 6.33 μmol mL^–1^ dimethyl sulfoxide (DMSO, Wako, 99.99%) as an internal standard. Calibration curves were generated by plotting standard methanol concentrations against the area ratio of methanol to DMSO. Due to the identical proton count in the methyl groups of CH_3_OH and CH_3_OOH, the same calibration curve was used for the quantitative analysis of both compounds.

For the HCHO-feeding controls, HCOOH/HCOO⁻ was identified by ^1^H NMR using the same liquid-phase sample preparation and a water-suppressed noesygppr1d acquisition at 500.13 MHz and 298 K (64 scans). The signal at δ ≈ 8.347 ppm was assigned to HCOOH/HCOO⁻, and DMSO at δ ≈ 2.623 ppm served as the internal reference. Approximate HCOOH/HCOO⁻ amounts were estimated semi-quantitatively by direct integration relative to DMSO, accounting for one proton for HCOOH/HCOO⁻ and six protons for DMSO. Because no dedicated HCOOH calibration or standard-addition recovery was performed, these estimates are not treated as calibrated quantitative values or used to claim a closed carbon balance. No CH_3_OH signal was detected near δ ≈ 3.3 ppm by ^1^H NMR under the stated analytical conditions.

The concentration of HCHO was determined using a colorimetric method.^[6]^ A reagent solution was prepared by dissolving 15 g of ammonium acetate, 0.3 mL of acetic acid, and 0.2 mL of pentane-2,4-dione in 100 mL of water. A mixture comprising 0.5 mL of the liquid product, 2.0 mL of water, and 0.5 mL of the reagent solution was incubated at room temperature for more than 5 hours. The concentration of HCHO was then assessed by measuring the absorption intensity at 412 nm using a UV-Vis absorption spectrometer, with the concentration determined from a standard curve.

H_2_O_2_ was tested by chemical titration method. 0.5 mL of the liquid product was mixed with 0.2 mL of 0.1 M KI solution and 50 µL 0.01 M H_32_Mo_7_N_6_O_28_ solution.^[7]^ After allowing the reaction to proceed for 10 min, the concentration of H_2_O_2_ was determined through analysing the absorbance of the solution at 352 nm using a UV-5500 PC spectrophotometer, following a five-fold dilution.

**Recycling procedure**

For the recycling experiment in Figure 1e, each cycle was conducted for 0.5 h under the standard conditions. After each reaction, the reactor was cooled and the catalyst was recovered by centrifugation, washed with deionised water, and dried before reuse. Because only 10 mg catalyst was used in each individual reaction, several parallel reactions were performed in the first cycle and the recovered catalysts were combined to provide sufficient material for subsequent cycles. For each subsequent cycle, 10 mg recovered catalyst was weighed and reused with 200 mL fresh water. The reactor was recharged with 3 bar O_2_ and 20 bar CH_4_ and operated under the same reaction conditions. Production rates were normalised to the 10 mg catalyst mass charged in each cycle.

**Additional HCHO-feeding controls**

For the HCHO-only controls, a commercial 16% (w/v) methanol-free aqueous formaldehyde solution (Thermo Scientific Pierce, Cat. No. 28908), prepared from paraformaldehyde and supplied methanol-free according to the manufacturer, was diluted with water before use, and an aliquot corresponding to 300 μmol HCHO was added to 200 mL H_2_O containing 10 mg ZnO or 2Au-ZnO, or to the corresponding catalyst-free blank. No purification or treatment other than dilution with water was applied before use. The reactor was charged with 3 bar O_2_ and 3 bar Ar and reacted at 150 °C under 365 nm irradiation (75 mW cm^-2^) and 500 rpm for 0.5 h. For the HCHO-addition experiment under methane-oxidation conditions, an aliquot of the diluted solution corresponding to 300 μmol HCHO was added to the standard 2Au-ZnO reaction containing 20 bar CH_4_ and 3 bar O_2_; all other conditions were unchanged. Residual HCHO was quantified by the acetylacetone colorimetric method described above. CH_3_OH and HCOOH/HCOO⁻ were evaluated by ^1^H NMR as described above, and gas products were analysed by GC. The results are presented as product amounts after 0.5 h (Figures S6 and S7).

**Temperature-dependent performance**

Bare ZnO and 2Au-ZnO were tested at 30, 70, 110, and 150 °C using 10 mg catalyst, 200 mL H_2_O, 3 bar O_2_, 20 bar CH_4_, 365 nm irradiation (75 mW cm^-2^), and a reaction time of 0.5 h. Product-resolved production rates and CH_3_OH selectivities are compared in Figure S10. C1 oxygenate production denotes the sum of the molecular production rates of CH_3_OOH, CH_3_OH, HCHO, and HCOOH where quantified.

**Product accounting and rate definitions**

Product amount or production is reported in μmol. Values in μmol h^-1^ are interval-average production rates, calculated as the product amount divided by reaction time. Mass-normalised production rates are reported in mmol g^-1^ h^-1^. For carbon-weighted totals, molecular product amounts are converted to carbon equivalents; C1 products contribute one carbon equivalent and molecular C_2_H_6_ contributes two. Quantified CO is included as a C1 product in the carbon-atom-based selectivity denominator.

The CH_3_OH selectivity was calculated on a carbon-atom basis from quantified carbon-containing products using Equation (1):

Selectivity of CH_3_OH = n(CH_3_OH) / [n(CH_3_OOH) + n(CH_3_OH) + n(HCHO) + n(HCOOH) + 2n(C_2_H_6_) + n(CO) + n(CO_2_)] × 100%	(1)

where nᵢ is the molecular molar amount of product i over the stated reaction interval. HCOOH is included where it is quantitatively determined. The factor of 2 is applied once to molecular C_2_H_6_ to convert it to carbon equivalents.

**Figure S1.** Proposed green and current industrial processes for CH_3_OH production from methane. Although the route via synthesis gas requires harsh conditions and involves two distinct steps, it is utilised to produce most CH_3_OH. The direct route using oxygen and water via photo-thermo cascade catalysis holds promise due to its high selectivity and efficiency in producing CH_3_OH under mild conditions.

**Figure S2.** (a) Illustration of the setup for photo-thermo cascade catalytic methane oxidation including a high-pressure batch reactor, a heating mantle and an LED lamp. (b) Details of the high-pressure batch reactor, including pressure gauge, thermocouple, and gas path.

**Figure S3.** The ^1^H NMR spectrum of the liquid product obtained from photo-thermo cascade catalytic methane oxidation to methanol over Au-ZnO. Reaction conditions: 10 mg photocatalysts, 200 mL water, 20 bar CH_4_, 3 bar O_2_, 150 °C, 365 nm LED (75 mW cm^–2^, illumination area of 12.56 cm^2^), 0.5 h reaction. DMSO was used as an internal standard with a concentration of 1.266 μmol mL^–1^.

**Figure S4.** The GC chromatogram of the gas products obtained from photo-thermo cascade catalytic methane oxidation to methanol over Au-ZnO. Reaction conditions: 10 mg photocatalysts, 200 mL water, 20 bar CH_4_, 3 bar O_2_, 150 °C, 365 nm LED (75 mW cm^–2^, illumination area of 12.56 cm^2^), 0.5 h reaction.

**Figure S5.** The CH_3_OH production rate and selectivity over Au-ZnO with different Au loading amounts at 30 °C. Reaction conditions: 10 mg photocatalyst, 200 mL H_2_O, 1 bar O_2_ + 20 bar CH_4_, 365 nm LED (75 mW cm^–2^), 30 °C, 0.5 h.

**Figure S6.** HCHO-feeding controls in the absence of CH_4_. Product amounts obtained from 300 μmol methanol-free HCHO over ZnO, 2Au-ZnO, and without catalyst. Conditions: 10 mg catalyst where applicable, 200 mL H_2_O, 300 μmol HCHO, 3 bar O_2_ + 3 bar Ar, 150 °C, 365 nm LED irradiation (75 mW cm^-2^), 500 rpm, and 0.5 h. In the catalyst-free blank, 293.6 μmol HCHO remained (~2.1% apparent loss).

Residual HCHO over ZnO and 2Au-ZnO was 191.5 and 183.2 μmol, corresponding to approximately 36.2% and 38.9% HCHO conversion. No CH₃OH signal was detected by ^1^H NMR under the stated analytical conditions. HCOOH/HCOO⁻ was identified by ^1^H NMR; the plotted HCOOH/HCOO⁻ amounts are semi-quantitative estimates from direct integration relative to DMSO and are not used to claim a closed carbon balance. CO_2_ was quantified by GC. These results disfavor productive sequential conversion of released HCHO to CH_3_OH under the tested conditions.

**Figure S7.** Effect of added HCHO on methane oxidation over 2Au-ZnO. Product amounts under standard CH_4_ oxidation conditions without and with 300 μmol added HCHO. Conditions: 10 mg 2Au-ZnO, 200 mL H_2_O, 3 bar O_2_ + 20 bar CH_4_, 150 °C, 365 nm LED irradiation (75 mW cm^-2^), 500 rpm, and 0.5 h. Adding HCHO did not increase CH_3_OH production (387.6 μmol without added HCHO and 368.7 μmol with added HCHO), whereas CO_2_ increased from 45.8 to 136.5 μmol.

**Figure S8.** Effect of CH_4_ pressure on methane oxidation over 2Au-ZnO. Production rates of CH_3_OOH, CH_3_OH, HCHO, C_2_H_6_, and CO_2_, together with CH_3_OH selectivity, at CH_4_ pressures of 1, 10, and 20 bar. Reaction conditions: 10 mg photocatalyst, 200 mL H_2_O, 3 bar O_2_, 365 nm LED irradiation (75 mW cm^-2^), 150 °C, and 0.5 h reaction time.

Increasing CH_4_ pressure from 1 to 20 bar raises the total C1 oxygenate production rate from approximately 55.9 to 819.5 μmol h^-1^ while maintaining high CH_3_OH selectivity.

**Figure S9.** Product production rates in control experiments over 2Au-ZnO without light, without CH_4_, or without catalyst. Common conditions: 200 mL H_2_O, 3 bar O_2_, 150 °C, 500 rpm, and 1 h. The standard gas feed contained 20 bar CH_4_ unless CH_4_ was omitted; 10 mg 2Au-ZnO was used unless the catalyst was omitted; 365 nm irradiation (75 mW cm^-2^; illuminated area 12.56 cm^2^) was applied unless light was omitted.

**Figure S10.** Temperature-dependent product formation over (a) bare ZnO and (b) 2Au-ZnO. Conditions: 10 mg catalyst, 200 mL H_2_O, 3 bar O_2_ + 20 bar CH_4_, 365 nm LED irradiation (75 mW cm^-2^), and 0.5 h at 30, 70, 110, or 150 °C. Production rates of CH_3_OOH, CH_3_OH, HCHO, C_2_H_6_, and CO_2_ are shown together with CH_3_OH selectivity.

The C1 oxygenate production rate over ZnO increases from approximately 99.2 μmol h^-1^ at 30 °C to 228.0, 370.9, and 795.7 μmol h^-1^ at 70, 110, and 150 °C, respectively. Bare ZnO changes from CH_3_OOH-rich at 30 °C to HCHO-rich at elevated temperature; at 150 °C, HCHO and CH_3_OH production rates are approximately 678.3 and 37.4 μmol h^-1^, and CH_3_OH selectivity is ~4.27%. In contrast, CH_3_OH selectivity over 2Au-ZnO increases from 34.4% at 30 °C to 86.0% at 150 °C. Heating therefore accelerates both systems, while Au produces a distinct methanol-selective thermal response.

**Figure S11.** Product amounts over 2Au-ZnO as a function of reaction time with 3 bar O_2_ and 20 bar CH_4_.

Figure S11 shows the time-dependent product amounts and CH_3_OH selectivity. During the first 30 min, the interval-average CH_3_OH production rate is ~800 μmol h^-1^. From 30 to 60 min, the interval-average rate decreases to ~44 μmol h^-1^ as CH_3_OH accumulates and competing secondary conversion becomes more important. Beyond 120 min, the net CH_3_OH amount changes little, whereas CO_2_ continues to increase. The accumulated product amounts therefore reflect both formation and subsequent consumption rather than the gross radical-generation rate.

**Figure S12.** Photo-thermo cascade catalytic methanol oxidation under (a) O_2_ and (b) O_2_ + CH_4_ atmosphere. Reaction conditions: 10 mg Au-ZnO, 200 mL water containing 400 μmol methanol, 3 bar O_2_ or 20 bar CH_4_ + 3 bar O_2_, 365 nm LED (75 mW cm^−2^), 150 °C, 0.5 h. reaction.

**Figure S13.** High-resolution Au 4f XPS spectra of the fresh and used Au-ZnO.

**Figure S14.** (a) TEM image of used Au-ZnO. (b) Au particle size distribution of used Au-ZnO. (c) High-resolution TEM image and (d) element mappings of used Au-ZnO.

**Figure S15.** TPD-MS profile of CH_3_OH over (a) ZnO and (b) 2Au-ZnO.

The CH_3_OH TPD-MS profiles reveal distinct adsorption/desorption behaviour on ZnO and Au-ZnO. In the 50-200 °C range, ZnO shows a broad molecular-methanol feature (m/z = 31 and 32) centred at 91 °C, whereas Au-ZnO shows a lower-intensity maximum at approximately 100 °C, indicating that Au changes the population and/or strength of weak methanol adsorption sites. At higher temperatures, both samples show methanol-derived fragments around 284-297 °C. The broad high-temperature tail on ZnO (approximately 380-600 °C; m/z = 44 and 16), assigned to strongly bound oxidised carbonaceous species, is substantially suppressed on Au-ZnO. These profile differences indicate that Au alters methanol retention and secondary transformation under the He-TPD protocol. Because the TPD experiment is performed under He during a temperature ramp, the profiles are interpreted as adsorption/desorption evidence rather than as reaction-condition desorption kinetics or a deep-oxidation threshold.

**Figure S16.** Temperature-dependent in situ DRIFTS spectra of ZnO and Au-ZnO under methane-oxidation conditions. Difference spectra (light minus dark at the corresponding temperature) of (a) ZnO at 30 °C, (b) ZnO at 150 °C, (d) Au-ZnO at 30 °C, and (e) Au-ZnO at 150 °C after 1, 2, 5, 10, and 20 min of 365 nm irradiation. Spectra are vertically offset for clarity; dashed lines mark the regions near 1570 and 1370 cm^-1^. (c,f) Time-dependent local peak heights near (c) 1570 cm^-1^ and (f) 1370 cm^-1^ after local linear baseline correction. Measurements used water-vapour-saturated CH_4_/O_2_ (CH_4_:O_2_ = 20:3).

At 150 °C, ZnO shows pronounced time-dependent accumulation near 1570 and 1370 cm^-1^, whereas the same accumulation is much weaker over Au-ZnO. The ~1570 cm^-1^ region is consistent with OCO/formate-like species, while the ~1370 cm^-1^ region can also contain CH_3_ bending contributions. Because these assignments overlap, the peak-height curves are comparative markers of net surface-species accumulation rather than unique intermediate fingerprints or calibrated coverage measurements. The contrasting temporal evolution therefore supports an Au-dependent redistribution of methane-derived surface intermediates at elevated temperature.

**Figure S17.** Product production rates over Al_2_O_3_ and Au-Al_2_O_3_ at 150 °C. Reaction conditions: 10 mg catalyst, 200 mL H_2_O, 3 bar O_2_ + 20 bar CH_4_, 365 nm LED (75 mW cm^-2^), 150 °C, and 0.5 h.

**Figure S18.** Product production rates over Au-Al_2_O_3_@ZnO at different reaction temperatures. Reaction conditions: 20 mg catalyst, 200 mL H_2_O, 3 bar O_2_ + 20 bar CH_4_, 365 nm LED (75 mW cm^-2^), and 0.5 h.

**Figure S19.** Adsorption energies of CH_3_* on Zn and O sites of the ZnO surface.

CH_3_* denotes a surface-bound methyl species. It binds more strongly to a Zn site than to an O site; therefore, subsequent calculations of CH_3_* transfer and C–O coupling were initiated from CH_3_* adsorbed on the Zn site.

**Figure S20.** DFT-calculated energy profile for the C-O coupling between CH_3_* adsorbed on a Zn site and OOH* adsorbed on a neighbouring Zn site on ZnO.

The reaction proceeds with an activation barrier of 1.44 eV, reflecting the intrinsically difficult coupling of CH_3_*; formation of the C-O bond requires substantial cleavage of the initial C-Zn bond to expose an unsaturated carbon centre for coupling with the O atom of OOH*.

**Figure S21.** Relative Arrhenius factor increase (normalized to 30 °C) for the C–O coupling and CH_3_* transfer pathways over Au-ZnO upon heating from 30 to 150 °C.

**Figure S22.** H_2_O_2_ production analysis using UV-vis absorption of the KI/ H_32_Mo_7_N_6_O_28_ solution in dark (before reaction) and under light irradiation (after reaction). The concentration of H_2_O_2_ can be determined by measuring the absorbance of the solution at 352 nm. Reaction conditions: 10 mg photocatalyst, 200 mL H_2_O, 3 bar O_2_ + 20 bar CH_4_, 365 nm LED (75 mW cm^–2^), 30 °C, 0.5 h.

**Figure S23.** The CH_3_OH production rate and selectivity over Au-CeO_2_ with different Au loading amounts at 30°C. Reaction conditions: 10 mg photocatalyst, 200 mL H_2_O, 1 bar O_2_ + 20 bar CH_4_, 365 nm LED (75 mW cm^–2^), 30 °C, 0.5 h.

**Figure S24.** The CH_3_OH production rate and selectivity over Au-TiO_2_ with different Au loading amounts at 30°C. Reaction conditions: 10 mg photocatalyst, 200 mL H_2_O, 1 bar O_2_ + 20 bar CH_4_, 365 nm LED (75 mW cm^–2^), 30 °C, 0.5 h.

**Table S1.** The photo-thermo cascade catalytic results in the feed gas of O_2_ and methane with different methanol concentrations over the Au-ZnO catalyst.

| Entry | Reaction time (h) | Methanol in the feedstock (µmol) | Methanol in the outlet of the reactor (µmol) | Formaldehyde in the outlet of the reactor (µmol) |
| --- | --- | --- | --- | --- |
| 1 | 0.5 | 0 | 394 | 11 |
| 2 | 1 | 0 | 416 | 18 |
| 3 | 2 | 0 | 436 | 20 |
| 4 | 0.5 | 200 | 439 | 23 |
| 5 | 0.5 | 400 | 438 | 69 |

**References**

1. X. Chen, Y. Li, X. Pan, D. Cortie, X. Huang, and Z. Yi, “Photocatalytic Oxidation of Methane over Silver Decorated Zinc Oxide Nanocatalysts,” *Nature Communications* *7*, no. 1 (2016): 12273, https://doi.org/10.1038/ncomms12273.

2. G. Kresse, and J. Furthmüller, “Efficiency of Ab-Initio Total Energy Calculations for Metals and Semiconductors Using a Plane-Wave Basis Set,” *Computational Materials Science* *6*, no. 1 (1996): 15–50, https://doi.org/10.1016/0927-0256(96)00008-0.

3. P. E. Blöchl, “Projector Augmented-Wave Method,” *Physical Review B* *50*, no. 24 (1994): 17953–17979, https://doi.org/10.1103/PhysRevB.50.17953.

4. J. P. Perdew, J. A. Chevary, S. H. Vosko, et al., “Atoms, Molecules, Solids, and Surfaces: Applications of the Generalized Gradient Approximation for Exchange and Correlation,” *Physical Review B* *46*, no. 11 (1992): 6671–6687, https://doi.org/10.1103/PhysRevB.46.6671.

5. G. Henkelman, B. P. Uberuaga, and H. Jónsson, “A Climbing Image Nudged Elastic Band Method for Finding Saddle Points and Minimum Energy Paths,” *The Journal of Chemical Physics* *113*, no. 22 (2000): 9901–9904, https://doi.org/10.1063/1.1329672.

6. H. Song, X. Meng, S. Wang, et al., “Direct and Selective Photocatalytic Oxidation of CH_4_ to Oxygenates with O_2_ on Cocatalysts/ZnO at Room Temperature in Water,” *Journal of the American Chemical Society* *141*, no. 51 (2019): 20507–20515, https://doi.org/10.1021/jacs.9b11440.

7. C. Hu, H. Huang, F. Chen, Y. Zhang, H. Yu, and T. Ma, “Coupling Piezocatalysis and Photocatalysis in Bi_4_ NbO_8_ X (X = Cl, Br) Polar Single Crystals,” *Advanced Functional Materials* *30*, no. 7 (2020): 1908168, https://doi.org/10.1002/adfm.201908168.
